# Supplementary material for: Psychometric properties of performance-based measures of physical function administered via telehealth among people with chronic conditions: A systematic review
Source: PLoS One. 2022 Sep 9;17(9):e0274349. doi: 10.1371/journal.pone.0274349 (PMC9462578; doi:10.1371/journal.pone.0274349)
Supplement: S2 Fig — (PDF) [file pone.0274349.s002.pdf]

S2 Fig Search Strategy

Filter 1: Target Population

(MH "Chronic Disease+") OR (TI (“chronic condition*” or “chronic disease*” or “chronic health” or “chronic illness*” or disability or “long?term condition*” or “long?term disease*” or “long?term illness*” or musculoskeletal or pain or arthritis or osteoarthritis or hip or knee or back or shoulder or elbow or wrist or ankle or foot or neck or neuro* or stroke or cva or “cerebrovascular accident” or “multiple sclerosis” or ms or Parkinson* or cardiac or cardiovascular or cardiorespiratory or “heart failure” or “heart disease” or chf or ccf or respiratory or pulmonary or copd or “chronic obstructive pulmonary disease” or asthma or cf or “cystic fibrosis” or “interstitial lung disease” or “pulmonary fibrosis”) OR AB (“chronic condition*” or “chronic disease*” or “chronic health” or “chronic illness*” or disability or “long?term condition*” or “long?term disease*” or “long?term illness*” or musculoskeletal or pain or arthritis or osteoarthritis or hip or knee or back or shoulder or elbow or wrist or ankle or foot or neck or neuro* or stroke or cva or “cerebrovascular accident” or “multiple sclerosis” or ms or Parkinson* or cardiac or cardiovascular or cardiorespiratory or “heart failure” or “heart disease” or chf or ccf or respiratory or pulmonary or copd or “chronic obstructive pulmonary disease” or asthma or cf or “cystic fibrosis” or “interstitial lung disease” or “pulmonary fibrosis”)

Filter 2: Construct

TX "physical function*" or "functional task*" or "functional capacity" or "physical capacity" or "exercise capacity" or "physical activit*" or "motor activit*" or "physical performance*" or "functional performance*" or "functional limitation*" or "physical limitation*" or "functional analysis" or "functional status" or “clinical outcome assessment*” or “clinical measurement*” or “physical examination test”

Filter 3: Measurement Instrument

TI (assessment* or evaluation* or outcome* or measure* or test* or instrument* or exam or examination* or tool* or analysis or observation*) OR AB (assessment* or evaluation* or outcome* or measure* or test* or instrument* or exam or examination* or tool* or analysis or observation*)

Filter 4: Context

TI (telehealth or tele-health or telerehabilitation or tele-rehabilitation or telerehab or telemedicine or tele-medicine or e-health or ehealth or mhealth or m-health or technolog* or digital or internet or online or virtual or remote) OR AB (telehealth or tele-health or telerehabilitation or tele-rehabilitation or telerehab or telemedicine or tele-medicine or e-health or ehealth or mhealth or m-health or technolog* or digital or internet or online or virtual or remote)

Filter 5: COSMIN Measurement Properties Filter

(MH “Psychometrics”) or (TI psychometr* or AB psychometr*) or (TI clinimetr* or AB clinimetr*) or (TI clinometr* OR AB clinometr*) or (MH “Outcome Assessment”) or (TI outcome assessment or AB outcome assessment) or (TI outcome measure* or AB outcome measure*) or (MH “Health Status Indicators”) or (MH “Reproducibility of Results”) or (MH

“Discriminant Analysis”) or ((TI reproducib* or AB reproducib*) or (TI reliab* or AB reliab*) or (TI unreliab* or AB unreliab*)) or ((TI valid* or AB valid*) or (TI coefficient or AB coefficient) or (TI homogeneity or AB homogeneity)) or (TI homogeneous or AB homogeneous) or (TI “coefficient of variation” or AB “coefficient of variation”) or (TI “internal consistency” or AB “internal consistency”) or (MH “Internal Consistency+”) or (MH “Reliability+”) or (MH “Measurement Error+”) or (MH “Content Validity+”) or “hypothesis testing” or “structural validity” or “cross-cultural validity” or (MH “Criterion-Related Validity+”) or “responsiveness” or “interpretability” or (TI reliab* or AB reliab*) and ((TI test or AB test) OR (TI retest or AB retest)) or (TI stability or AB stability) or (TI interrater or AB interrater) or (TI inter-rater or AB inter-rater) or (TI intrarater or AB intrarater) or (TI intra-rater or AB intrarater) or (TI intertester or AB intertester) or (TI inter-tester or AB inter-tester) or (TI intratester or AB intratester) or (TI intra-tester or AB intra-tester) or (TI interobserver or AB interobserver) or (TI inter-observer or AB inter-observer) or (TI intraobserver or AB intraobserver) or (TI intra-observer or AB intra-observer) or (TI intertechnician or AB intertechnician) or (TI inter-technician or AB inter-technician) or (TI intratechnician or AB intratechnician) or (TI intra-technician or AB intra-technician) or (TI interexaminer or AB interexaminer) or (TI inter-examiner or AB inter-examiner) or (TI intraexaminer or AB intraexaminer) OR (TI intra-examiner or AB intra-examiner) or (TI intra-examiner or AB intraexaminer) or (TI interassay or AB interassay) or (TI inter-assay or AB inter-assay) or (TI intraassay or AB intraassay) or (TI intra-assay or AB intra-assay) or (TI interindividual or AB interindividual) or (TI inter-individual or AB inter-individual) OR (TI intraindividual or AB intraindividual) or (TI intra-individual or AB intra-individual) or (TI interparticipant or AB interparticipant) or (TI inter-participant or AB inter-participant) or (TI intraparticipant or AB intraparticipant) or (TI intra-participant or AB intra-participant) or (TI kappa or AB kappa) or (TI kappa’s or AB kappa’s) or (TI kappas or AB kappas) or (TI repeatab* or AB repeatab*) or (TI responsive* or AB responsive*) or (TI interpretab* or AB interpretab*)
