# Supplementary material for: Psychometric properties of performance-based measures of physical function administered via telehealth among people with chronic conditions: A systematic review
Source: PLoS One. 2022 Sep 9;17(9):e0274349. doi: 10.1371/journal.pone.0274349 (PMC9462578; doi:10.1371/journal.pone.0274349)
Supplement: S1 Table — (PDF) [file pone.0274349.s003.pdf]

S1 Table. Criteria for Good Measurement Properties

Property	Rating	Quality Criteria
Reliability	+	ICC/weighted kappa ≥ 0.70
	?	ICC or weighted kappa not reported
	-	ICC/weighted kappa < 0.70
Criterion validity	+	Correlation with gold standard ≥ 0.70 OR AUC ≥ 0.70
	?	Not all information for '+' reported
	-	Correlation with gold standard < 0.70 OR AUC < 0.70

'+' sufficient rating, '?' indeterminate rating, '-' insufficient rating *(Terwee et al. 2007; Prinsen et al. 2016)*

AUC= area under the curve, ICC= intraclass correlation coefficient
