# Supplementary material for: Psychometric properties of performance-based measures of physical function administered via telehealth among people with chronic conditions: A systematic review
Source: PLoS One. 2022 Sep 9;17(9):e0274349. doi: 10.1371/journal.pone.0274349 (PMC9462578; doi:10.1371/journal.pone.0274349)
Supplement: S2 Table — (PDF) [file pone.0274349.s004.pdf]

S2 Table. Instructions on the use of the modified GRADE approach (Mokkink et al. 2017; Prinsen et al. 2018; Terwee et al. 2018)

Quality of Evidence	Lower if
High	Risk of bias<br>-1 Serious<br>-2 Very serious<br>-3 Extremely serious<br><br>Inconsistency<br>-1 Serious<br>-2 Very Serious<br><br>Imprecision<br>-1 total n=50-100<br>-2 total n<50<br><br>Indirectness<br>-1 Serious<br>-2 Very serious
Moderate	
Low	
Very Low	

n=sample size

Risk of Bias	Downgrading for Risk of Bias
No	There are multiple studies of at least adequate quality, or there is one study of very good quality available
Serious	There are multiple studies of doubtful quality available, or there is only one study of adequate quality
Very serious	There are multiple studies of inadequate quality, or there is only one study of doubtful quality available
Extremely serious	There is only one study of inadequate quality available
