# Supplementary material for: Psychometric properties of performance-based measures of physical function administered via telehealth among people with chronic conditions: A systematic review
Source: PLoS One. 2022 Sep 9;17(9):e0274349. doi: 10.1371/journal.pone.0274349 (PMC9462578; doi:10.1371/journal.pone.0274349)
Supplement: Attachment — Submitted filename: Response to Reviewers.docx [file pone.0274349.s005.docx]

**Response to Reviewers**

We thank the Reviewers for their constructive feedback which has substantially improved the manuscript. We have revised the manuscript and provided a response to each point raised by the Reviewers below.

**Reviewer #1**

***1) Use of the COSMIN Checklist: the references to the COSMIN checklist link to recommendations for patient reported outcome measures (PROM). The use of COSMIN methodology seems reasonable for these performance based (not self-reported) tools, but needs to be justified in the methods and discussed in the study limitations***

Thank you for raising this concern. We have amended the references to the COSMIN checklist which now refer to the checklist for performance-based measurement instruments as opposed to the original checklist for patient-reported measurement instruments. While the methodology is similar across both checklists we have ensured we have adhered to the performance-based measurement instrument guidelines throughout our revised manuscript and this is now reflected in our updated reference list. The reference for the PROM checklist is still included in instances where the performance-based checklist advised users to refer to the PROM checklist for guidance (e.g. when using the modified GRADE approach one must refer to the original PROM guidelines)

***2) The COSMIN user manual that is referenced recommends a 10 step process for systematic review of measurement properties. For a comprehensive review such as this one I would expect statements describing the methodology and findings for:

*Content validity including tool development
*Internal structure
*Remaining measurement properties (reliability, measurement error, criterion validity, hypothesis testing + responsiveness)
*Interpretability and feasibility

While some of these domains may not be directly applicable for the tools you have identified, and others may have no available data, I would suggest this should be acknowledged in your methods and then considered in your discussion as deviations from the COSMIN method.***

Thank you for highlighting this point.

To adhere to our pre-registered protocol we have included this in our eligibility criteria in the methods section: “Measurement properties: In our pre-registered protocol, we highlighted studies must have reported one or more of the psychometric measurement properties from the COSMIN taxonomy (1). For studies examining the validity of the measurement instrument administered via telehealth, the comparator was a face-to-face administration of the same measurement instrument. Since the comparator was always face-to-face administration of the same measure, when extracting data from the selected studies the measurement properties of interest were reliability, measurement error and criterion validity. Therefore, the remaining measurement properties outlined in the COSMIN taxonomy including other forms of validity and interpretability were not considered to be outcomes of interest in this review.”

Given the eligibility criteria and aims of our review, the outcomes of interest were reliability, measurement error and criterion validity. For this reason we did not report findings for the remaining measurement properties. This was highlighted in our results section in the following line: “No studies reported data on measurement error or responsiveness”

We also included the following in the limitations section within the discussion:

“Although Cox et al. (2) reported the feasibility of the three minute step test administered via telehealth and Hwang et al. (3) reported some information regarding the number and nature of technical issues encountered during telehealth administration of the 6MWT, TUGT and grip and pinch strength, there was limited information reported in the included studies regarding the interpretability and feasibility of the included measures when administered via telehealth. As noted in the eligibility criteria, this review was concerned with examining the criterion validity of measures administered via telehealth when compared to face-to-face administration of the same measure. Therefore, other types of validity, such as content and construct validity, were not reported in this review. Due to the eligibility criteria and aims of this review, the outcomes of interest were reliability, measurement error and criterion validity. For this reason, we followed COSMIN recommendations for evaluating reliability, measurement error and criterion validity but a priori did not choose to evaluate other types of validity, internal structure, interpretability and feasibility.”

***3) Study construct/measurement instrument selection: you describe the construct of interest as "performance-based physical function" and your measurement instruments of interest as "performance measures". Do you mean that the type of physical function you are interested in relates to performance, or that the measurement tools require an evaluation of performance (i.e. not self-reported). I would suggest that you use performance-based for one of these uses but not both.***

Thank you for highlighting this potential source of confusion. We were interested in reviewing measurement instruments that required an evaluation of performance to measure physical function. We refer to these measures as ‘performance-based measures of physical function’. We have edited the relevant lines to include this.

***4) Evaluation of measurement properties. It would be helpful to have a description of the validity/reliability domains and the thresholds applied to decide if measurement properties of satisfactory/indeterminant and inadequate to guide a reader who is unfamiliar with COSMIN through your methods***

We have now included the following information in the ‘Evaluation of the measurement properties reported in the included studies’ section of the Methods, to provide greater detail and clarity for readers:

“For the reliability domain, inter-rater and intra-rater reliability were evaluated by comparing the scores for the measure when administered via telehealth between different raters and also when administered by the same rater at two different time points. As recommended, a threshold of 0.70 on the intraclass correlation (ICC) or weighted kappa was used to evaluate the reliability of the measure administered via telehealth (4). If the correlation was ≥o 0.70 the reliability received a sufficient rating. If the ICC or weighted kappa was not reported it received an indeterminate rating for reliability. The reliability of the measure was rated as insufficient if the ICC or weighted kappa score was < 0.70.

For the criterion validity domain, the measure administered via telehealth was compared to the same measure administered in a face-to-face environment. A correlation of 0.70 with the reference standard (4), which for the purpose of this review was the measure administered in a face-to-face environment, was the threshold. The validity of the measure was rated as sufficient if the correlation was ≥ 0.70. The validity was rated as indeterminate if correlations were not reported. The validity was rated as insufficient if the correlation with the reference standard was < 0.70”

We have included a more detailed description of the domains and thresholds applied to evaluate the measurement properties, as well as highlighting the thresholds used, in Table S1.

***5) 'Gold standard': The authors have used a gold standard of face to face measurement for criterion validity. I would argue that this is more likely to be a reliability measurement, or even a hypothesis test of construct validity as it is possible that an invalid test for physical function may yield similar values if administered face to face or via telemedicine. While this is an important measurement property I do not feel that it is sufficient robust to use as gold standard.***

Thank you for raising this concern. The aim of our review was to examine the psychometric properties of performance-based measures of physical function administered via telehealth among people with chronic health conditions. Adhering to our protocol, when examining the validity of telehealth assessments the comparator for the review was the face to face administration of the same measure. We acknowledge your concerns regarding the use of the term ‘gold standard’ to describe the face to face measures and we have edited our manuscript to now refer to the face to face administration of the measure as the ‘reference standard’.

We acknowledge your point that a poor test might be equally ‘invalid’ in both environments, and thus appear inappropriately valid, we believe that criterion validity is the most appropriate term to use to describe these comparisons. We have also discussed the validity and reliability of these tests in a face-to-face setting in our discussion section, and these data do not suggest any major reason for concern. Our use of this term is in agreement with other authors who conducted a similar review when examining the validity of objective clinical examinations in any healthcare field when administered by student clinicians via telehealth compared to an in-person format (5). We have also discussed the validity and reliability of these tests in other settings too, and these data do not suggest any major reason for concern.

***6) Study characteristics: I would be interested to know where the patients were located for these validation studies. Were these patients at home (akin to telemedicine during the pandemic) or patients brought to a clinic or laboratory to participate in the study. This would have significant feasibility implications for patient-at-home telemedicine rehabilitation as discussed in the background.***

Thanks for raising this useful point. We have added any information from the included studies regarding patient location in the ‘Telehealth Environment’ column of Table 1. Many studies involved the assessor being located in a separate room to the patient within the same building. Although the particular building was often not explicitly described in the studies, it is presumed this was a clinical or research setting as it did not state in any studies that the patient was located at home. All information regarding patient and assessor location that was included in the relevant studies has now been included in Table 1.

***7) Methodological quality of studies: I found this section unclear as study quality (risk of bias) seemed to blend with the adequacy of measurement properties reported. In addition, I am confused as to how studies could be selected for reporting criterion validity but then downgraded for not reporting correlation. How did these studies compare their telemedicine to face to face measurements?***

In recognition of the complexity of the COSMIN system, we have expanded upon the explanation provided in the revised manuscript. The terminology used to rate the **quality of studies** is different to the terminology used to evaluate the **measurement properties**. The quality of studies is rated as very good, adequate, doubtful or inadequate. In contrast, the measurement properties are evaluated as sufficient, indeterminate or insufficient. These classifications are based on the COSMIN methodology and have been used throughout this review (4).

On the specific point raised regarding ‘how studies could be selected for reporting criterion validity but then downgraded for not reporting correlation’, some of the included studies reported the validity of telehealth assessments when compared to face-to-face assessment by reporting the mean difference between the measures, but without including correlations. In such a situation the quality of the study is classified as ‘inadequate’ since neither correlations nor the area under the receiver operating curve are reported (6). We believe this is a limitation of the COSMIN methodology, which has now been discussed in our results in the ‘Methodological Quality of Included Studies’ section and again in the discussion.

***8) Summary of findings: You report unidimensionality in this table but this has not been described in the background or methods. How did you assess unidimensionality and is this relevant for single-item scores?***

Thank you for highlighting this concern. This was included in error and has been removed from the Table 3 Summary of Findings

***9) Discussion lines 323-377 are a well written description of the results and I feel would be better situated in your results section.***

Thank you, we have now situated the majority of this information in the results section. However we feel that some of this information relating to the comparison of our findings with the wider (non-telehealth) literature is more relevant for our discussion section and therefore we have situated this information in the discussion section as follows:

“The findings of sufficient reliability and criterion validity when administered via telehealth mirror that reported when many measures are administered among chronic populations in a face-to-face environment, including the 6MWT (7, 8) and grip strength test (9, 10), as well as the Berg Balance Scale, TUGT and the Step Test (11-15).”

***10) Clinical implications: Given the very low quality of evidence available I do not think you can conclude that any measures have adequate reliability and validity in this study. I think the implication should be toned down to several performance measures may have satisfactory reliability and validity.***

We have edited this section to state that “several performance-based measures of physical function across different domains (e.g. exercise capacity, strength and balance) may have satisfactory reliability and criterion validity when used in a telehealth environment” and have highlighted that this evidence is of low-very low quality”. We have tried in this section to reflect both (i) the low quality of evidence and (ii) the fact that the COSMIN approach does not recognise and reward studies for determining validity using mean differences, even when the value of arbitrary correlation cut-offs such as 0.7 is not without issues.

***11) Strengths and limitations: you discuss limitations in the studies included but not the potential limitations and mitigations from your study design and execution. These include:

Use of the COSMIN checklist for non-PROM tools
Not undertaking study screening and full text extraction in duplicate
Not seeking indirect evidence of measurement properties from other populations***

***Use of the COSMIN checklist for non-PROM tools***: The point regarding the use of the COSMIN checklist for non-PROM tools is similar to point 1). As per our earlier response the checklist for performance-based measurement instruments was used to guide this review and the references have been updated to reflect this. In other words, what we have done is aligned with the COSMIN approach, but the guidance for this comes from both performance-based and non-performance based guidance.

***Not undertaking study screening and full text extraction in duplicate***: We have now acknowledged the point regarding screening and full text extraction in the limitations section as follows: “As previously stated, two independent reviewers screened a sample of 30% of abstracts and relevant full texts to determine eligibility. As good agreement was achieved on the 30% sample, the remaining screening process was not performed in duplicate.”

***Not seeking indirect evidence of measurement properties from other populations***: Thank you for highlighting this concern. In our protocol we specified the focus of this review to examine the psychometric properties of performance-based measures of physical function administered via telehealth among people with chronic health conditions. Therefore, as we aimed to explore a specific population, we did not seek to include evidence from other populations. We have highlighted this in our limitations and acknowledge that this may limit our findings:

“Further evidence may have been identified from studies of post-operative populations, such as individuals post total knee arthroplasty (16). However, these were excluded as pain and disability levels immediately post-operatively, and how much these change relatively quickly, are quite different from other chronic conditions where physical function may be more stable over time.”

**Reviewer #2**

***1- Correct the " COSMIN (Consensus-Based Standards for the Selection of Health Measurement Instrument)" phrase in lines 28 and 29 to " Consensus-Based Standards for the Selection of Health Measurement Instrument (COSMIN)". Correct other spellings like this throughout the manuscript.***

This change is accepted and addressed in the relevant lines.

***2- Indicate which authors are the reviewers mentioned on lines 163-169.***

The initials of the relevant authors have now been included in the relevant lines.

***3- Write the reference representations of the studies mentioned in Table 1 and Table 2.***

This change is accepted and addressed in Table 1 and Table 2.

***4- Specify the definitions of abbreviations such as n, SD in Table 1.***

Thank you, we have specified the abbreviations included in Table 1 in the legend below the table.

**Reviewer #3**

***1- Search strategy should be updated.***

We have updated the search and present the revised search strategy results in the manuscript, tables and figures. In short, no new studies met the eligibility criteria.

***2- Abstract: authors should report specific chronic conditions, telehealth environment and tools tested in the results and make conclusion on the quality of the current evidence supporting any investigate tool, if so. Besides, it is important to raise the potential issue related to the external validity (only high Human Development Index/HDI settings?).***

We have now included additional details regarding the chronic populations, telehealth environment and settings of the included studies. We have not outlined the measurement instruments included due to word count limitations on the abstract, however we have outlined the domains of physical function that the measures assess. We have also edited the abstract to meet the 300 word limit.

The results section of the abstract now reads as follows:

“Five articles met the eligibility criteria. These included patients with Parkinson’s Disease (n=2), stroke (n=1), cystic fibrosis (n=1) and chronic heart failure (n=1). Fifteen performance-based measures of physical function administered via videoconferencing were investigated, spanning measures of functional balance (n= 7), other measures of general functional capacity (n= 4), exercise capacity (n= 2), and functional strength (n= 2). Studies were conducted in Australia (n=4) and the United States (n=1). Reliability was reported for twelve measures, with all twelve demonstrating sufficient inter-rater and intra-rater reliability. Criterion validity for all fifteen measures was reported, with eight demonstrating sufficient validity and the remaining seven demonstrating indeterminate validity. No studies reported data on measurement error or responsiveness.”

We have also highlighted this potential issue related to external validity in the limitations section within the discussion.

***3- Methods, selection of studies: Only 30% of abstracts and potential full texts were independently assessed by two independent reviewers in the selection of studies. Add it as a limitation in the discussion section.***

This change is accepted and the following has been included in the limitations section in the discussion: “There were also some limitations to be acknowledged. As previously stated, two independent reviewers screened a sample of 30% of abstracts and relevant full texts to determine eligibility. As good agreement was achieved on the 30% sample, the remaining screening process was not performed in duplicate.”

***4- Methods, page 10. Please add reference for the following statement: “As per COSMIN guidance, in order to demonstrate ‘sufficient’ validity the measure must
198 demonstrate >0.70 correlation with the ‘gold standard’.”.***

Thank you, this change is accepted and addressed.

***5- Methods, synthesis: please clarify if it is appropriate to include studies not comparing with face to face.***

To adhere to our protocol we have now included the following line in our eligibility criteria: “For studies examining the validity of the measurement instrument administered via telehealth, the comparator was be a face-to-face administration of the same measurement instrument.”

Therefore it is not appropriate to include studies that explored the validity of measures administered via telehealth unless they compared with face-to-face administration of the same measure.

***6- Methods: criteria to downgrade due to quality of the evidence in each domain of the GRADE approach should be specified. Was it conducted by two independent and trained reviewers? How did they resolve potential disagreements, if so?***

The GRADE approach was used by two independent reviewers and this has now been outlined in the methods section. However neither of the reviewers received formal training in this method and this has now been acknowledge in the limitations as follows: “Although the quality of the summarised evidence was rated using the modified GRADE approach by two independent reviewers, neither of these reviewers were formally trained in the use of this method.”

Detailed instructions outlining the criteria to downgrade in each domain of the GRADE approach is outlined in S2 table. The following was added to the methods section within the manuscript:

“Detailed instructions on the use of the modified GRADE approach to grade the quality of the summarised evidence can be found in S2 Table. The starting point assumed that the summarised result was of high quality and was downgraded by one, two or three levels depending on the risk of bias. The summarised result was further downgraded depending on the inconsistency, imprecision and indirectness associated with the summarised result as appropriate.”

Further information regarding the criteria to downgrade the evidence for the imprecision domain was also added to the methods section:

“For imprecision, the evidence was downgraded one level if the sample size was 50-100 individuals. If the sample size was less than 50 individuals the evidence was downgraded two levels.”

***7- Results: further description on characteristics of the samples is needed because it may be a potential external validity to be discussed. Participants: from the community? rural?, Socioeconomic status?, Cognitively tested?, and able to use mobile, computer, both?***

We have included as much information as was available from the included studies regarding the sample characteristics, however, there is limited information regarding the same and this has now been acknowledged in the limitations section as follows:

“There was limited information reported regarding the characteristics of the samples in the included studies in relation to aspects such as socioeconomic status, cognitive status and technological literacy. These factors could potentially impact the external validity of the findings.”

***8- Results, Table 1: Hwang et al 2017 conducted synchronous videoconferencing as all the other included studies? Please clarify in the Table.***

Yes, Hwang conducted synchronous videoconferencing and this has been clarified in Table 1.

***9- Discussion: authors should revise discussion section to accommodate comments.***

As per the above responses the limitations section of the discussion has been revised to accommodate for the above comments.

***10- References: replace reference 29 to “LB Mokkink, M Boers, CPM van der Vleuten, LM Bouter, J Alonso, DL Patrick, HCW de Vet, CB Terwee. COSMIN Risk of Bias tool to assess the quality of studies on reliability or measurement error of outcome measurement instruments: a Delphi study. BMC Medical Research Methodology. 2020;20(293).”. Is is the specific reference for performance-based outcome measures (PerFOMs).***

Thank you, this change is accepted and addressed.

***11- References: reference for risk of bias should be “Lidwine B. Mokkink, Maarten Boers, CPM van der Vleuten, LM Bouter, Jordi Alonso, Donald L Patrick, HCW de Vet, CB Terwee. COSMIN Risk of Bias tool to assess the quality of studies on reliability or measurement error of outcome measurement instruments: a Delphi study. BMC Medical Research Methodology. 2020;20(293).” Besides, please revise if it was used correctly by two independent reviewers with disagreements resolved by consensus or a third reviewer.***

Thank you, we have corrected this reference. We followed the COSMIN recommendations for assessing the methodological quality of included studies which is as follows: “We recommend that each study is assessed by two independent reviewers, and that they come to consensus” (4). We have edited the methods section to reflect this.

**Reviewer #4**

***Abstract-
Results: please re-order according to frequency***

Thank you, this change is accepted and addressed.

***What about other types of validity such as content, face, and construct validity? Can you add a bit about these too?***

This is similar to point 2 made by Reviewer #1. As per our protocol, studies examining the validity of measures administered via telehealth were only included if they compared these measures to the same measures administered via telehealth. Therefore the outcome of interest was criterion validity and we did not report other types of validity. This has been added to the abstract as follows: “No studies reported data on measurement error or responsiveness.”

***Also, you discuss reliability, which type of reliability was that?***

This change is accepted and the line now reads: “Reliability was reported for twelve of the measures, with all twelve demonstrating sufficient inter-rater and intra-rater reliability”

***Introduction-
This section was extremely well-written.***

Thank you.

***Line 59- “The ageing nature of our population..” I agree this is true in many Western countries but not necessarily everywhere, therefore, I suggest changing our.***

This change is accepted and addressed. This line now reads: “The ageing nature of the Western world and the increasing prevalence of chronic conditions presents a significant socioeconomic burden and will continue to persistently challenge health care services (3, 4)”.

***Methods-
Authors did a meticulous work in this section as well.***

Thank you.

***Line 142- for the constructs, please add a few examples***

This change is accepted and examples of strength and balance have been added.

***Line 154- "However, since the comparator was always face-to-face administration of the same measure, when extracting data from the selected studies the only relevant properties were reliability, measurement error and criterion validity."
This is great explanation but can go into your discussion or limitation. I think here under measurement properties you must say that you planned to include any measurement property reported in the papers.***

Thank you for this comment. This is similar to previous concerns made by the Reviewers. Please also see our responses to their comments. To adhere to our pre-registered protocol we specified that the review aimed to examine the validity of assessments administered via telehealth when compared to face-to-face administration of the same measure. Therefore given our eligibility criteria and aims,

the outcomes of interest were reliability, measurement error and criterion validity. For this reason we did not report findings for the remaining measurement properties.

We also included the following in the limitations section within the discussion:

“Although Cox et al. (2) reported the feasibility of the three minute step test administered via telehealth and Hwang et al. (3) reported some information regarding the number and nature of technical issues encountered during telehealth administration of the 6MWT, TUGT and grip and pinch strength, there was limited information reported in the included studies regarding the interpretability and feasibility of the included measures when administered via telehealth. As noted in the eligibility criteria, this review was concerned with examining the criterion validity of measures administered via telehealth when compared to face-to-face administration of the same measure. Therefore, other types of validity, such as content and construct validity, were not reported in this review. Due to the eligibility criteria and aims of this review, the outcomes of interest were reliability, measurement error and criterion validity. For this reason, we followed COSMIN recommendations for evaluating reliability, measurement error and criterion validity but a priori did not choose to evaluate other types of validity, internal structure, interpretability and feasibility.”

***Line 160- I think you can connect using just 'or' instead of and/or. This is discouraged in academic writing.***

Thank you, this change is accepted and addressed.

***Results-
Line 252- minor grammatical error, use were instead of was.***

Thank you for highlighting this, this change is accepted and addressed.

***Line 254- I suggest adding something like ‘…Or other types of validity’***

Thank you, this change is accepted and addressed.

***Line 255- Same comment as in the abstract, please re-order based on the frequency of the tests, or alphabetically.***

This change is accepted and addressed.
 ***Discussion-
Line 326- Consider re-writing: This evidence reflects the evidence supporting***

This was addressed and now states: The 6MWT demonstrated sufficient reliability and criterion validity when administered via telehealth. This result reflects the reliability and validity of this measure when administered among chronic populations in a face-to-face environment (7, 8).

***Line 379- remove 'of' in following of PRISMA***

Thank you, this change is accepted and addressed.

***Line 400- I would be cautious to say that '....have satisfactory reliability and validity when used in a telehealth environment.' especially with validity since you only found data for criterion validity and other types were not addressed.***

This is similar to the concern raised by Reviewer #1. As per the previous response we have adjusted these lines and they now state:

“Encouragingly, several performance-based measures of physical function across different domains (e.g. exercise capacity, strength and balance) may have satisfactory reliability and criterion validity when used in a telehealth environment. Furthermore, the psychometric properties of these measures appears similar to that reported for the same measures when used in a face-face context. This should reassure clinicians that using performance-based measures of physical function via telehealth is possible. However, this evidence is of low-very low quality and there is a significant lack of information regarding the measurement error and responsiveness of these measures. Furthermore, information regarding the interpretability and feasibility of the included measures was very limited (2, 3).”

References

1.	Mokkink LB, Terwee CB, Patrick DL, Alonso J, Stratford PW, Knol DL, et al. The COSMIN checklist for assessing the methodological quality of studies on measurement properties of health status measurement instruments: an international Delphi study. Quality of life research. 2010;19(4):539-49.

2.	Cox NS, Alison JA, Button BM, Wilson JW, Holland AE. Assessing exercise capacity using telehealth: a feasibility study in adults with cystic fibrosis. Respiratory Care. 2013;58(2):286-90.

3.	Hwang R, Mandrusiak A, Morris NR, Peters R, Korczyk D, Russell T. Assessing functional exercise capacity using telehealth: Is it valid and reliable in patients with chronic heart failure? Journal of Telemedicine and Telecare. 2017;23(2):225-32.

4.	Mokkink LB, Boers M, Van Der Vleuten C, Bouter LM, Alonso J, Patrick DL, et al. COSMIN Risk of Bias tool to assess the quality of studies on reliability or measurement error of outcome measurement instruments: a Delphi study. BMC medical research methodology. 2020;20(293).

5.	Felthun JZ, Taylor S, Shulruf B, Allen DW. Assessment methods and the validity and reliability of measurement tools in online objective structured clinical examinations: a systematic scoping review. Journal of Educational Evaluation for Health Professions. 2021;18.

6.	Mokkink LB, De Vet HC, Prinsen CA, Patrick DL, Alonso J, Bouter LM, et al. COSMIN risk of bias checklist for systematic reviews of patient-reported outcome measures. Quality of Life Research. 2018;27(5):1171-9.

7.	Uszko-Lencer NH, Mesquita R, Janssen E, Werter C, Brunner-La Rocca H-P, Pitta F, et al. Reliability, construct validity and determinants of 6-minute walk test performance in patients with chronic heart failure. International journal of cardiology. 2017;240:285-90.

8.	Singh SJ, Puhan MA, Andrianopoulos V, Hernandes NA, Mitchell KE, Hill CJ, et al. An official systematic review of the European Respiratory Society/American Thoracic Society: measurement properties of field walking tests in chronic respiratory disease. European Respiratory Journal. 2014;44(6):1447-78.

9.	Bobos P, Nazari G, Lu Z, MacDermid JC. Measurement properties of the hand grip strength assessment: a systematic review with meta-analysis. Archives of physical medicine and rehabilitation. 2020;101(3):553-65.

10.	Lamers I, Kelchtermans S, Baert I, Feys P. Upper limb assessment in multiple sclerosis: a systematic review of outcome measures and their psychometric properties. Archives of physical medicine and rehabilitation. 2014;95(6):1184-200.

11.	Hill KD, Bernhardt J, McGann AM, Maltese D, Berkovits D. A new test of dynamic standing balance for stroke patients: reliability, validity and comparison with healthy elderly. Physiotherapy Canada. 1996;48(4):257-62.

12.	Blum L, Korner-Bitensky N. Usefulness of the Berg Balance Scale in stroke rehabilitation: a systematic review. Physical therapy. 2008;88(5):559-66.

13.	Jácome C, Cruz J, Oliveira A, Marques A. Validity, reliability, and ability to identify fall status of the Berg Balance Scale, BESTest, Mini-BESTest, and Brief-BESTest in patients with COPD. Physical therapy. 2016;96(11):1807-15.

14.	Christopher A, Kraft E, Olenick H, Kiesling R, Doty A. The reliability and validity of the timed up and go as a clinical tool in individuals with and without disabilities across a lifespan: A systematic review: Psychometric properties of the timed up and go. Disability and Rehabilitation. 2021;43(13):1799-813.

15.	Mesquita R, Wilke S, Smid DE, Janssen DJ, Franssen FM, Probst VS, et al. Measurement properties of the Timed Up & Go test in patients with COPD. Chronic respiratory disease. 2016;13(4):344-52.

16.	Cabana F, Boissy P, Tousignant M, Moffet H, Corriveau H, Dumais R. Interrater agreement between telerehabilitation and face-to-face clinical outcome measurements for total knee arthroplasty. Telemedicine and e-Health. 2010;16(3):293-8.
